# The old man’s friend

**DOI:** 10.1186/s41479-018-0052-7

**Published:** 2018-07-25

**Authors:** Ger T. Rijkers, Stephen I. Pelton

**Affiliations:** 1 Science Department, University College Roosevelt, P.O. Box 94, 4330 AB Middelburg, The Netherlands; 2 Boston Medical Center, Boston University Schools of Medicine and Public Health, Boston, USA

## Editorial

The term “old man’s friend” is often used when referring to pneumonia. Searching for it on Google yields 16,400 results in 0.33 s for this combination. The term is attributed to William Osler, who in the first edition of his book *The Principles and Practice of Medicine* (1892) wrote:

In children and in healthy adults the outlook is good. In the debilitated, in drunkards and in the aged the chances are against recovery. So fatal is it in the latter class [i.e. the elderly] that it has been termed the natural end of the old man [1].

In the 9th edition, published after Osler himself already died (in 1919 from pneumonia at the age of 70 years [2]), this excerpt was rephrased as “.. . one may say that to die of pneumonia is almost the natural end of old people” [3]. But that was 100 years ago. Fortunately, a lot changed for the better in the century that followed.

Can old men be friends with pneumonia? Is it possible to like, or even be in love with, a disease? As a patient, no! As a biomedical researcher, yes, that certainly is possible. The study of the interaction between *Streptococcus pneumoniae* and the human immune system can be in the focus of one’s scientific interest for 38 years. For a pediatrician, out of the great number of inherited or acquired diseases affecting infants and children, it is not uncommon to specialize in pneumonia. The biomedical researcher in the above example is Professor Ger Rijkers, the pediatrician is Professor Stephen Pelton, and together we will be taking the reins from Professor Allan Cripps AO as the new co-Editors-in-Chief of *Pneumonia*.

The history of *Pneumonia*, the journal, that is, began in April 2006, when the 5th International Symposium on Pneumococci and Pneumococcal Disease was held in Alice Springs, Central Australia [4]. In the outdoors, *Musca vetustissima*, as well as representatives of the more than 10,000 other species in the order of Diptera, were trying to get into every orifice of the ISPPD participants. Indoors, *Streptococcus pneumoniae*, as well as other representatives of micro-organisms that can cause pneumonia, were trying to get into the brains of the meeting’s participants. While in this environment, Allan Cripps had the epiphany to start a new journal focused solely on pneumonia [5]. But why? As he explained in a later interview:

By forming a journal with an exclusive focus on pneumonia, we are dedicated to establishing an international forum for pneumonia in the broadest context and as a means for bringing together knowledge related to pathogenesis, treatment and prevention of pneumonia [6].

“It is with great pleasure that I welcome you to pneumonia” were Allan’s opening words in the inaugural editorial of *Pneumonia* in 2012 [5]. Today, it is with great pleasure that we take over from Allan. To draw on the words of one of *Pneumonia*’s Editorial Board members, Professor Ron Dagan, Allan has done a wonderful job taking the Journal from conception to birth through to its adolescence. It is now our time to guide *Pneumonia* to maturity.

*Pneumonia* was and will continue to be a peer-reviewed, online, open access journal. It was and will continue to be dedicated to providing an international forum for pneumonia in the broadest context and a means for bringing together knowledge related to the pathogenesis, treatment and prevention of pneumonia. To that end, we will publish original research articles, case studies, reviews, critical commentaries, correspondence, highlights and news on all aspects of pneumonia. We will, in this era of rapidly evolving communication concepts, also consider novel ways to disseminate the science of pneumonia. We will also—in this era of misinformation, fake news and science denial—stand firm for science and for scientific integrity.

Vaccination has led to the disappearance of smallpox from the world; therefore, a journal with exclusive focus on smallpox would not be viable today. Will *Pneumonia* (the journal) share the same fate? Although vaccines are available and continue to improve, currently and for the foreseeable future pneumonia (the disease) remains a global burden with high incidence in the youngest and oldest of our globe’s inhabitants and new pathogens are continually emerging [7, 8]. For the foreseeable future, *Pneumonia* the journal is here to stay.

### Trends and challenges in the fight against pneumonia

There are a number of important themes, challenges and areas of interest in the ongoing fight against pneumonia that we look forward to disseminating in the Journal. The differences in disease burden between adults and children, especially infants, is highlighted by the epidemiology that reveals most pneumonia occurs in healthy children and yet is associated with high mortality rates linked to socially determinants. The highest incidence rates and mortality are found in India, South East Asia and Africa, but the Global Alliance for Vaccines and Immunisation (GAVI) has enabled introduction of pneumococcal-conjugate vaccines that are expected to have a substantial impact on the current disease burden. We look forward to new insights into the respiratory microbiome, such as whether the microbiome can predict those infants at risk for pneumonia, chronic pulmonary disease, recurrent wheezing and, if so, whether modulation of the microbiome can change these outcomes.

The distribution of pathogens causing pneumonia still remains challenging. We look forward to new diagnostic approaches that will deconstruct the pathogens that cause pneumonia. The limitation of current diagnostic tools is best demonstrated by the discord between vaccine probe studies and those that assign a more limited role to the pneumococcus in studies of pneumonia. It is hoped that molecular tools may reveal pulmonary pathogens by deciphering the specifics of the host response to bacteria compared to viruses.

One of the most common cause of respiratory disease in children remains respiratory syncytial virus (RSV). Despite decades of effort, no proven treatment or prevention strategies have emerged. We appear to be on the precipice and look forward to new and expanded treatments for respiratory syncytial virus infection as well as an RSV vaccine that will further reduce the burden of respiratory disease.

## Conclusion

Today, pneumonia still affects many ‘old’ men. Medical progress made since William Osler’s time has resulted in survival rate for hospitalized pneumonia that now sits above 90–95%. However, longer-term mortality is high. The reasons for this are still largely unknown. A hypothesis from the editors of *Pneumonia*? Perhaps chronic inflammation leading to silent progression of cardiac disease is an underlying mechanism.

We, as the new co-Editors-in-Chief, standing on the shoulders of Allan Cripps, welcome submissions on any of the themes indicated above, or other aspects of pneumonia that could ultimately lead to a better understanding of the disease, and thus better diagnosis, prevention and treatment.
